# Splenectomy for Splenomegaly

**Published:** 1913-12

**Authors:** J. A. Nixon

**Affiliations:** Physician to the Bristol Royal Infirmary


### SPLENECTOMY FOR SPLENOMEGALY.

BY

J. A. Nixon, M.B. Cantab., F.R.C.P.,

*Physician to the Bristol Royal Infirmary.*

There are many conditions in which enlargement of the spleen occurs. Often, too, the spleen enlarges before any other morbid changes have declared themselves. If then a case presents itself with no other sign than splenomegaly, it is impossible to forecast the ultimate developments which may overtake such a case. In the endeavour to classify a doubtful case and determine the type to which it most nearly conforms valuable time may be lost. Experience shows that simple splenomegaly is only too frequently the first stage of some serious or fatal malady. According to Osler [1] the ultimate outlook of simple splenomegaly is bad, and there is only one radical cure—removal of the spleen. The results of splenectomy are so favourable, and the fate of splenomegalics with unremoved spleens so unsure, that it seems nowadays unjustifiable to temporise and waste valuable time in administering drugs or "trying X-rays" in the hope that the disease may be arrested.

Apart from leukæmia, there is an increasing volume of evidence that the proper treatment for splenomegaly of doubtful origin and type is splenectomy. Stein, [2] discussing Banti's disease and allied conditions, says, "The various attempts to overthrow or to dissociate the pathological and clinical complex constructed by Banti have so far been unsuccessful, and the brilliant results obtained by surgical intervention will always stand." Similarly Goebel [3] and Bondi advise that early extirpation of the spleen should always be performed in Banti's disease. Burghard [5] considers that there is good prospect of cure, and that the risks of operation are slight if the spleen is removed in the early stages of Banti's disease before the onset of the terminal hepatic cirrhosis and ascites. William Mayo [6] has recently described the results of operation on eighteen cases of splenic anæmia (including Banti's disease): the results were gratifying. "if the disease is uncomplicated, splenectomy may be expected to cure the patient." He advocates this operation in other splenic enlargements of toxic or microbic origin, and states that in primary tuberculosis of the spleen early splenectomy may effect a cure, quoting in support of this statement Franke's ten cases operated upon with seven cures. Malarial spleens may, according to Mayo, be extirpated with advantage, as well as movable and wandering spleens: "rotation of the spleen leads to death or splenectomy." Lott [7] published in 1912 an account of recovery following splenectomy for this accident. In the primary splenomegaly of Gaucher (endothelioma of the spleen) early splenectomy is followed by recovery.[8] Descarpentries[9] has successfully operated in a case of "icterus splenomegalique." Stengel[10] strongly advocates operation in cases of splenomegaly with hæmolytic jaundice. In fact, the only conditions in which splenectomy for splenomegaly seems inadvisable are (*a*) leukæmia, where the operation is invariably fatal; (*b*) alcoholic or syphilitic cirrhosis of the liver with splenomegaly; (*c*) in splenomegaly associated with primary pylethrombosis.

The accompanying table gives the results of 708 cases of splenectomy up to the year 1908, summarised by Johnston.[11]

| Lesion or Disease. | Cases. | Recovered. | Died. |
|---|---|---|---|
| Idiopathic hypertrophy .. .. .. .. | 74 | 53 | 21 |
| Idiopathic hypertrophy, ectopic spleen | 64 | 50 | 6 |
| Idiopathic hypertrophy, twisted pedicle | 27 | 19 | 8 |
| Malarial hypertrophy .. .. .. .. .. | 149 | 111 | 38 |
| Malarial hypertrophy, ectopic spleen .. | 40 | 39 | 1 |
| Malarial hypertrophy, twisted pedicle .. | 12 | 10 | 2 |
| Splenic anæmia .. .. .. .. .. .. | 61 | 49 | 12 |
| Cysts, hydatid .. .. .. .. .. .. .. | 23 | 19 | 4 |
| Cysts, non-parasitic .. .. .. .. .. .. | 19 | 19 | 0 |
| Leukæmia .. .. .. .. .. .. .. .. | 49 | 6 | 43 |
| Tuberculosis .. .. .. .. .. .. .. | 10 | 8 | 2 |
| Sarcoma .. .. .. .. .. .. .. .. | 12 | 9 | 3 |
| Abscess .. .. .. .. .. .. .. .. | 9 | 8 | 1 |
| Miscellaneous .. .. .. .. .. .. .. | 13 | 11 | 2 |
| Wounds and injuries .. .. .. .. .. | 150 | 99 | 51 |
| Total .. .. .. | 708 | 514 | 194 |
| Per cent. .. .. | | 72.6 | 27.4 |

The following case illustrates the insignificant disturbances which may attend excision of the spleen for simple splenomegaly.

Alice R., aged 14, a factory girl, came under my care in the Bristol Royal Infirmary in July, 1912, complaining of pain across the upper part of the abdomen, of an intermittent character, chiefly localised to the left side, extending into the groin and down the left leg. Occasionally the pain was of a distinctly colicky type. Until a year previously the girl had always been strong and healthy, but she then suddenly "became anæmic," and was obliged to leave school. A typical attack was described as follows:—"Three weeks or a month ago she woke up in the morning with severe stabbing pain across the top of the stomach and down the left side into the left leg. The pain was very severe and lasted all the morning. The next morning the same thing happened, and it recurred every other day up to the time of admission. Violent exercise or jolting in a tramcar would bring on the pain. Sometimes she felt sick with pain, but had never actually vomited. There had been no appearance of blood in the urine, nor any trouble with micturition." The patient was a well-grown girl, pale, anæmic and sallow-complexioned, with numerous flecks of pigment or large freckles about the skin. No stigmata of congenital syphilis.

*Abdomen.*—No visible abnormality. On palpation a considerable area of resistance was felt in the left hypochondrium, caused by a smooth, somewhat convex swelling which moved on respiration, extended beneath the ribs above and had a well-defined margin inferiorly; no definite edge could be felt and no notch made out.

The tumour was dull on percussion, and the dulness extended into the spleen area. No band of colon resonance could be found passing over the front of the tumour. The maximum size of the swelling was about four fingers' breadth below the ribs.

*Cutaneous hyperæsthesia.*—The skin was exceedingly sensitive to a light touch or firm pressure over the whole left side of the abdomen and the whole of the left leg, where a pinch, which was scarcely felt on the right leg, caused exquisite pain. There was also on the abdomen a well-defined zone of tactile hyperæsthesia and hyperalgesia, corresponding with the distribution of the tenth dorsal nerve from the spinal processes to the mid-line in front, with the usual spots of tenderness to deep pressure.

*Vertebral column.*—There was some apparent rigidity of the spine in the lower dorsal region, with prominence of the spinous processes. But no particular spine was unduly prominent or deviated. Percussion of the spine and jarring of the heels caused pain to be felt radiating round the left side of the body down towards the groin. On bending backwards or forwards there was always an inclination of the body towards the right side.

Examination of the abdomen and spine caused considerable pain, which lasted for two or three hours afterwards.

All reflexes were normal, and no other abnormality of the nervous system was discovered.

*Heart and lungs.*—Healthy in all respects.

*Lymphatic glands.*—No enlarged glands.

*Urine.*—No abnormal appearance or quantity, sp. gr. varied between 1010–1022. Once or twice a faint trace of albumen was present, but nothing else abnormal. No deposit. No casts.

*Temperature.*—Never raised above normal.

*Catamenia.*—Not yet commenced.

*Skiagrams.*—Showed a uniform dense shadow in the left hypochondriac region, suggestive of an enlarged spleen. No spinal caries or renal calculus shown.

*Blood examination.*—Hæmoglobin, 50 per cent.; red corpuscles, 5,000,000; white corpuscles, 12,600. Differential leucocyte count: polymorphonuclears, 55 per cent.; large mononuclears, 14 per cent.; small mononuclears, 26 per cent.; eosinophiles, 5 per cent. No abnormal cells seen.

*Family history.*—No member of the family had suffered from any similar condition, and the history was unimportant.

*Operation.*—The patient was so far incapacitated by her pain that an exploratory laparotomy was performed by Mr. Bush on July 31st, 1912.

The tumour was found to be an enlarged spleen. There was no evidence of renal enlargement, spinal disease, or psoas abscess. The spleen was excised. The gastro-splenic omentum was so short that its division and tying off caused great difficulty, and it was only by exercising great care and ligaturing the omentum in three sections that the inclusion of part of the stomach wall in the pedicle was avoided.

The spleen on removal measured 7 inches in its long axis and 3 inches in its shorter. It looked massive and about three times the average size for the age of the patient. Otherwise its appearance was normal.

The opinion was expressed that the symptoms could not have been caused by the splenic enlargement.

*Pathological report.*—Professor Walker Hall reported that the enlargement was due to simple hyperplasia of normal spleen tissue.

The patient made an uneventful recovery, except for considerable pain, tympanites and a pulse-rate of 140 the night after the operation.

The hyperæsthesia and girdle pain had completely disappeared by the next day. The whole of the symptoms were relieved, and the patient has remained quite well up to the present time (Aug., 1913). There has been no enlargement of lymphatic glands and no pain in the long bones.

*Blood examination.*—Repeated blood counts have been performed upon the patient. The only changes which appear to have resulted from the splenectomy were a temporary increase in eosinophiles, and the appearance for a few days only of a large number of cells of a transitional type. These contained horseshoe or multipartite nuclei, but the protoplasm showed no granular staining. They resembled large mononuclear cells with multipartite nuclei.

There was also an increase of lymphocytes at the expense of the polymorphonuclears.

| | Hæmo-globin. | Reds. | Whites. | Differential leucocyte count. | | | | | |
|---|---|---|---|---|---|---|---|---|---|
| | | | | Poly-nuclear. | Mononuclear. Small. | Mononuclear. Large. | Eosino-phile. | Transi-tional. | |
| | % | | | % | % | % | % | % | |
| 23rd July, 1912 .. | 50 | 5,000,000 | 12,600 | 55 | 26 | 14 | 5 | — | |
| 31st ,, ,, .. | | | | Operation. | | | | | |
| 3rd August ,, .. | 70 | 3,800,000 | 8,700 | 65 | 27 | 7 | 1 | — | |
| 4th ,, ,, .. | — | — | — | 74 | 18 | 6 | 2 | — | |
| 5th ,, ,, .. | — | — | — | 63 | 15 | 17 | 5 | — | |
| 7th ,, ,, .. | — | — | — | 56 | 22 | 14 | 8 | — | |
| 8th ,, ,, .. | 80 | 5,000,000 | 10,200 | 57 | 25 | 10 | 6 | 2 | |
| 9th ,, ,, .. | — | — | — | 29 | 48 | 10 | 8 | 5 | |
| 10th ,, ,, .. | — | — | — | 36 | 47 | 11 | 5 | 1 | |
| 16th ,, ,, .. | 80 | 4,500,000 | 9,600 | 34 | 50 | 10 | 5 | 1 | |
| 25th ,, ,, .. | 75 | 4,000,000 | 11,600 | 41 | 40 | 10 | 6 | 3 | 2 Basophiles. |
| 16th Oct., ,, .. | 80 | 4,800,000 | 10,000 | 41 | 36 | 17 | 6 | Some transitionals. | |
| 2nd Dec., 1913 .. | 80 | 4,200,000 | 9,000 | 52 | 37 | 7 | 3 | — | |

This case serves as yet another illustration of the impunity with which the spleen may be removed. The blood changes were of the usual kind and transitory. The low colour index was at once raised after operation and remained permanently raised. There was a relative increase in lymphocytes, and a temporary appearance of a transitional type of leucocyte. There was an eosinophilia before the operation which was scarcely affected. No cause could be assigned to this eosinophilia. It will be interesting to watch the subsequent progress of the patient, and observe whether any of the later phenomena which sometimes attend splenomegaly befall her in her spleenless state.

*Later Note.*

Dec. 2nd, 1913.—The girl has attended the Tuberculosis Dispensary for the past two months, with suspicious signs at both apices and a recurrence of the pain in the back accompanied by a girdle-hyperæsthesia. Her temperature is persistently raised, and I am again suspicious of spinal caries.

REFERENCES.

[1] Osler, *Principles and Practice of Medicine*, 8th Ed., 1912, p. 888.
[2] Stein, *Am. J. M. Sc.*, 1912, cxliv. 856.
[3] Goebel, *München Med. Wchnschr.*, 1912, lix. 840.
[4] Bondi, *Wien. Klin. Wchnschr.*, 1912, xxv. 327.
[5] Burghard, *A System of Operative Surgery*, iii. 1909, 45.
[6] Mayo, *Surg. Gyn., and Obst.*, 1913, xvi. 233.
[7] Lott, *Am. J. Obst.*, 1912, lxvi. 985.
[8] Downes, *Med. Rec.*, 1913, lxxxii. 697 ; *Am. Surg.*, 1913, lvii. 935.
[9] Descarpentries, *Écho Méd. du Nord.*, 1911, xv. 617.
[10] Stengel, *Progressive Med.*, 1912, ii. 266.
[11] Johnston, *Ann. Surg.*, 1908, xlviii. 50.

